# Serial Static Infrared Thermography for Intraoperative and Postoperative Surface-Temperature Monitoring of Free-Flap Skin Islands in Paediatric Craniofacial Reconstruction: A Technical Feasibility Study

**DOI:** 10.3390/jcm15207775

**Published:** 2026-10-09

**Authors:** Agnieszka Szurko, Zuzanna Sokołowska, Agnieszka Kiełboń, Anna Olejnik, Armand Cholewka, Maciej Borowiec, Tadeusz Morawiec, Michael R. Markiewicz, Martin Starosta, Anna Krzykowska, Krzysztof Dowgierd

**Affiliations:** 1 Faculty of Science and Technology, University of Silesia in Katowice, Bankowa 12, 40-007 Katowice, Poland; agnieszka.kielbon@us.edu.pl (A.K.); armand.cholewka@us.edu.pl (A.C.); 2 Department of Clinical Paediatrics, Head and Neck Surgery Clinic for Children and Young Adults, University of Warmia and Mazury, Zołnierska 18a Street, 10-561 Olsztyn, Poland; zuzanna.sokolowska@wssd.olsztyn.pl (Z.S.); olej.ania@gmail.com (A.O.); borowiecmaciej@gmail.com (M.B.); anna.krzykowska02@gmail.com (A.K.); krzysztofdowgierd@gmail.com (K.D.); 3 Department of Dental Surgery, Faculty of Medical Sciences in Zabrze, Medical University of Silesia, 40-055 Katowice, Poland; tmorawiec@sum.edu.pl; 4 Department of Oral and Maxillofacial Surgery, School of Dental Medicine, University at Buffalo, The State University of New York, 3435 Main Street, Buffalo, NY 14214, USA; mrm25@buffalo.edu; 5 Department of Dentistry, Faculty of Medicine, University of Ostrava, Syllabova 19, 703 00 Ostrava, Czech Republic; martin.starosta@osu.cz

**Keywords:** infrared thermography, microsurgical anastomosis, free flap monitoring, intraoperative imaging, paediatric craniofacial reconstruction, ALT flap, perfusion assessment, thermal imaging

## Abstract

**Background/Objectives:** This single-patient retrospective study evaluated the technical feasibility of serial infrared thermography (IRT) for intraoperative and postoperative surface-temperature documentation of free-flap skin islands and its descriptive concordance with clinical and vascular assessments. **Methods:** A 9-year-old boy with Nager syndrome undergoing bilateral temporomandibular joint reconstruction and soft-tissue reconstruction with an anterolateral thigh free flap divided into two skin islands was monitored intraoperatively and postoperatively. Thermograms were acquired using a FLIR T1020 camera (emissivity 0.98) under documented environmental conditions. Intraoperative and postoperative observations were interpreted separately, without normalisation between settings. Mean temperatures within manually delineated regions of interest and inter-island differences were analysed retrospectively and compared descriptively with documented clinical assessment, Doppler ultrasonography, and computed tomography angiography (CTA) findings. **Results:** Pallor prompted Doppler ultrasonography, followed by CTA; these showed absent right-perforator flow and non-visualisation of that perforator, respectively. The skin did not bleed after puncture. Thermography was performed after these tests and was not taken into account when making the decision about reoperation. IRT demonstrated temperature changes in both skin islands. At 49 h after the initial flap measurement, the mean surface temperature was 26.6 °C in the right skin island and 32.7 °C in the left (absolute difference, 6.1 °C). A focal area of relatively increased surface temperature “hot spot” was identified retrospectively within the viable left skin island; this qualitative observation was exploratory and was not based on a prespecified thermal threshold. **Conclusions:** Serial IRT was feasible and provided contactless thermal information retrospectively concordant with multimodal perfusion assessment. These findings are hypothesis-generating and do not establish earlier detection, diagnostic accuracy, or a universal thermal threshold; larger prospective studies are required.

## 1. Introduction

The clinically decisive question in microvascular free-flap reconstruction is whether emerging perfusion compromise can be recognised early enough to permit effective revision. Monitoring should therefore be repeatable, rapid, and feasible both in the operating room and during postoperative surveillance.

Thermographic approaches can be broadly distinguished into static and dynamic protocols. Static thermography records surface-temperature distribution at discrete time points without a controlled thermal stimulus, whereas dynamic infrared thermography (DIRT) commonly evaluates tissue rewarming following a standardised cooling challenge [1,2]. In the present study, serial static thermography was used; no controlled cooling–rewarming challenge was applied.

Infrared thermography (IRT) is a non-invasive, non-contact imaging technique that records infrared radiation emitted by the skin to map its surface temperature. It enables rapid image acquisition and repeated observations without ionising radiation, providing a practical means of documenting spatial temperature differences and their changes over time. In reconstructive surgery, IRT has been explored for flap surveillance and the identification of perfusion abnormalities [3,4]. However, available reports remain heterogeneous in acquisition conditions, region-of-interest definitions, and interpretations of thermal differences. Intraoperative serial data are particularly limited, and no validated temperature cutoff for free-flap viability can currently be inferred from the present evidence base.

In this study, infrared thermography (IRT), which records skin-surface temperature without tissue contact, was evaluated retrospectively alongside documented clinical and vascular assessments rather than used as an independent diagnostic test.

Nager syndrome (NS) (OMIM 154400; ORPHA 245), also known as Nager acrofacial dysostosis and AFD1 limb-facial dysostosis, is a rare congenital disorder associated with multiple craniofacial abnormalities. The most common symptoms include micrognathia, cleft palate, limb abnormalities, mandibular hypoplasia, and temporomandibular joint (TMJ) abnormalities [5,6].

Due to the wide range of defects associated with Nager syndrome, patients require coordinated, multidisciplinary, and long-term treatment. The first step is to improve breathing and feeding abilities [6]. Early mandibular advancement, including distraction osteogenesis, may be required in childhood to address severe airway obstruction, whereas definitive orthognathic surgery and secondary craniofacial reconstruction are usually performed during adolescence, after skeletal maturity is reached [7].

The free flap transplant has become a widely recognised and utilised method in reconstructive medicine for various pathologies occurring throughout the entire body. Numerous scientific studies conducted on large groups of patients who have undergone different procedures demonstrate that it is a technique with extremely high treatment efficacy, while simultaneously ensuring the restoration of function and aesthetics of the operated areas [3,8,9,10,11,12,13]. However, it should be emphasised that free flap failure, although rare, can be a catastrophic complication. The need for reoperation and removal of necrotic tissue leads to prolonged hospitalisation, and subsequent reconstruction is associated with a deterioration in expected functional and aesthetic outcomes [9].

Reconstructions using free flaps are currently the gold standard of treatment for complex deformities. Although microsurgical techniques have been extensively analysed in the adult population, we still have limited data on paediatric patients and young adults, especially regarding factors that determine treatment success. Due to the different physiological conditions and developmental stages characteristic of childhood, the healing process and tissue response to surgical intervention require individualisation and the use of specific surgical strategies [14].

The aim of this single-patient retrospective technical feasibility analysis was to evaluate the technical feasibility of serial infrared thermography for intraoperative and postoperative monitoring of free-flap skin-island perfusion. The analysis also assessed retrospective descriptive concordance between thermographic patterns and documented clinical assessment, Doppler ultrasonography, and computed tomography angiography.

## 2. Materials and Methods

### 2.1. Study Design

This was a single-patient retrospective observational technical feasibility analysis based on serial thermographic measurements acquired intraoperatively and postoperatively during the patient’s treatment as an additional procedure alongside standard clinical care. The index reconstruction was performed on 19 August 2025. The quantitative thermographic analysis presented here was performed retrospectively after ethical approval was granted on 14 July 2026. Diagnosis and treatment decisions, including the decision to reoperate, were based on clinical examination, Doppler ultrasonography and computed tomography angiography (CTA), not on thermography. The thermal findings were subsequently compared with the findings documented during treatment. The two monitored skin islands originated from the same free flap and are therefore anatomically and biologically linked; repeated measurements from the two islands were not treated as independent biological replicates. The analysis focused on technical feasibility, serial temperature behaviour, and descriptive concordance with established clinical and imaging assessments.

### 2.2. Patient Context and Surgical Indication

The patient was a 9-year-old boy with Nager syndrome phenotype. He was born at term after an uneventful first pregnancy to non-consanguineous parents. He was delivered by caesarean section with an Apgar score of 7/8/8.

The patient has undergone a series of complex operations over the past few years. In December 2016, a tracheostomy, Nissen fundoplication, and gastrostomy were performed to improve respiratory and digestive functions and prevent gastric reflux. From February 2017 to November 2018, two mandibular distractions were performed to improve airway patency. Those procedures were complicated by the development of TMJ ankylosis.

In April 2019, bilateral temporomandibular joint ankylosis release was performed, which improved airway patency and enabled tracheostomy decannulation. In the following years, follow-up polysomnography (PSG) examinations were performed. The last PSG performed in April 2024 revealed severe obstructive sleep apnoea with an AHI of 46.3. Due to this result, the patient was qualified for urgent surgery to improve airway patency. In May 2024, the patient underwent another mandibular distraction osteogenesis. In the following months, progressive limitation of mouth opening was observed, and follow-up computed tomography (CT) revealed recurrence of TMJ ankylosis. The most recent surgery, performed on 19 August 2025, consisted of bilateral TMJ ankylotic block resection and reconstruction with custom total alloplastic TMJ prostheses. In addition, facial soft-tissue reconstruction was carried out using a myocutaneous anterolateral thigh (ALT) free flap, resulting in improvements in both facial aesthetics and function.

### 2.3. Surgical Procedure

The procedure consisted of bilateral resection of temporomandibular joint ankylotic blocks and reconstruction with patient-specific total alloplastic temporomandibular joint prostheses. A myocutaneous anterolateral thigh (ALT) free flap was harvested from the left thigh and divided into two skin islands based on separate perforators arising from the same vascular pedicle for bilateral preauricular and submandibular soft-tissue reconstruction. Microvascular arterial and venous anastomoses were performed to the facial vessels on the right side using Synovis GEM coupler devices. The total procedure lasted approximately 8 h. All flap-monitoring times are referenced to the initial thermographic acquisition (0 h), obtained during flap dissection, before pedicle division. The clinical stages corresponding to the intraoperative measurements are summarised in the Section 3.

The procedure was performed under general anaesthesia with nasotracheal intubation, supplemented with local infiltration of 0.5% lidocaine with epinephrine (1:250,000). Mean arterial blood pressure was 90/50 mmHg during resection of the TMJ ankylosis with TMJ prostheses implantation and flap dissection, and 110/70 mmHg from the moment of performing vascular anastomosis until the end of the operation.

Bilateral preauricular incisions were made along pre-existing scars, extended submandibularly and temporally. After careful dissection through scarred tissues, the mandibular ramus and ankylotic blocks of both temporomandibular joints were exposed. The main trunk of the facial nerves was identified and preserved. Patient-specific cutting guides were positioned on the mandibular ramus and temporal regions, fixation screw holes were drilled, and osteotomies were performed to resect the ankylotic blocks according to the preoperative plan. The components of the patient-specific temporomandibular joint prostheses were positioned and fixed, achieving stable bilateral reconstruction.

Simultaneously, a myocutaneous anterolateral thigh (ALT) free flap was harvested from the left thigh. Perforators were identified, and the pedicle based on the descending branch of the lateral circumflex femoral artery was dissected. The flap was divided into two skin paddles based on separate perforators, temporarily repositioned at the donor site, covered with gauze moistened with saline solution, and maintained under these conditions throughout recipient site preparation, with serial temperature monitoring.

At the 3 h time point, the vascular pedicle was divided, marking the onset of flap ischemia, and the flap was subsequently transferred to the recipient site. The arterial and venous microvascular end-to-end anastomoses to the facial vessels on the right side were performed using Synovis GEM coupler devices (artery 1.5 mm; vein 3.0 mm) and had been completed by the 4 h assessment. At 5 h, subsequent reperfusion of the flap was visible in both skin colour evaluation and the presence of bleeding from the flap margin. Two skin islands were inset bilaterally to reconstruct the preauricular and submandibular soft tissues, while the de-epithelialized portion was used to cover and protect the patient-specific temporomandibular joint prostheses, providing stable soft-tissue coverage and separation from surrounding scarred tissues. The 7 h measurement was obtained during assessment of the flap after completion of the final inset. Drains were placed, and all wounds were closed in layers. Dressings were applied to the skin islands and donor site.

### 2.4. Infrared Thermography

All measurements were performed in accordance with the ‘Standards and Protocols in Clinical Imaging Thermography’ introduced by the International Academy of Clinical Thermology (IACT), which served as the methodological reference for image acquisition and quality control in the present study.

The patient underwent thermographic imaging during surgery in the operating theatre. The protocol used serial static thermography rather than a standardised cooling–rewarming challenge; temperature changes during routine surgery were not treated as a controlled dynamic test. No standardised cooling–rewarming challenge or other controlled thermal stimulus was applied, and rewarming kinetics were not analysed. At each scheduled time point, an experienced physicist with clinical thermography experience took three thermographic images. During recording, external heat sources and reflective surfaces were avoided, and the previously specified distance between the camera and the examined surface, as well as proper imaging geometry, were maintained. If tubes or other clinical equipment degraded the image sharpness or hindered the clear delineation of the skin island boundaries, such images were considered technically unsuitable for quantitative ROI analysis. For quantitative analysis, one image from each time point was used, selected based on the best technical quality and the clearest visibility of the skin island boundaries. Repeated recordings aimed to ensure at least one image suitable for interpretation and were not used to assess technical repeatability. The exact timing of image acquisition and analysis was not prospectively recorded. Thermographic imaging did not delay or interfere with standard clinical care.

Standard monitoring of the transferred right and left skin islands included a blind Doppler probe, Minidop ES-100VX (Hadeco, Inc., Kawasaki, Japan), and clinical assessment consisting of skin colour evaluation and puncture testing with assessment of bleeding response. Infrared thermographic images were acquired as an additional procedure for comparison with the standard clinical and vascular findings. Doppler ultrasonography was performed intraoperatively before division of the flap into two skin islands and subsequently during postoperative monitoring during assessment episodes corresponding to the reported thermographic time points. All Doppler examinations were performed by the surgical team. An audible signal was recorded as present when detected with the probe; failure to detect a signal was reported as “no audible signal obtained”. The complete monitoring schedule and applied assessment methods are presented in Table 1.

#### 2.4.1. Camera Positioning and Acquisition Geometry

Thermographic measurements were acquired using a FLIR T1020 thermal imaging camera (FLIR Systems, Inc., Wilsonville, OR, USA) with a native infrared resolution of 1024 × 768 pixels and an emissivity setting of 0.98. According to the manufacturer, the thermal sensitivity (noise-equivalent temperature difference, NETD) is <20 mK at 30 °C. NETD describes the detector’s ability to resolve small temperature differences and should not be interpreted as absolute temperature-measurement accuracy. The camera underwent annual calibration at an authorised service centre. In addition, monthly quality-control verification was performed using a blackbody reference source with a known, stable temperature (model BB-G, manufactured by IRTSys, Warszawa, Poland). During these verification checks, the measured blackbody temperature remained within the manufacturer-specified accuracy range of ±2% of the reference value. No out-of-range verification result was recorded during the period relevant to this study.

During thermographic examinations of skin islands within the facial recipient sites, the camera was positioned laterally relative to the patient’s head, directed directly at the cheek containing the examined skin island. The right and left sides were assessed separately, and each measurement involved a single lateral surface of the face, not a frontal view. The distance between the camera and the skin island was maintained at approximately 50 ± 5 cm. The front lens plane was set approximately parallel to the local plane of the surface of the examined skin island. The central optical axis, passing through the centre of the lens and the centre of the skin island, was approximately oriented perpendicular to the local plane of its surface. Thus, the optical axis was aligned with the local normal to the skin surface, corresponding to an angle of about 90° relative to the plane of the skin island’s surface.

At each corresponding time point of thermographic assessment, both skin islands were examined while maintaining the same previously specified conditions of thermographic acquisition and exposure of the tested surfaces. The patient’s head position during subsequent thermographic acquisitions was kept as consistent as possible, and both skin islands remained exposed and accessible for imaging. The entire surface of each skin island remained in contact with the atmospheric air. At image acquisition, the skin island surfaces were not covered with dressings; only narrow strips of dressing were applied at the suture sites, while the entire surface subjected to thermographic evaluation remained uncovered. The dressing arrangement was adapted to the shape of the island and did not cover the areas being thermographically assessed.

In the case of corresponding measurements of the right and left skin islands, acquisition geometry, camera-to-surface distance, surface exposure, and lighting were kept as consistent as clinically feasible. The same lateral imaging orientation was used for both sides, with the optical axis approximately perpendicular to the local surface plane of the respective skin island.

Conditions directly related to the course of the surgical procedure, including the character of the surgical field, the method of surgical manipulation, tissue handling, and other circumstances arising from the current stage and course of the operation, were not controlled or standardised for thermographic measurements. They resulted from the actual course of the surgical procedure and the clinical activities undertaken during it, which could not be modified to achieve uniform measurement conditions, nor was it appropriate to do so. Thermographic examinations were carried out as part of the accepted clinical procedure, without interfering with the course of the operation. Standardisation, therefore, concerned the conditions of the thermographic acquisition itself and the exposure of the evaluated skin surfaces, while intraoperative conditions reflected the real clinical circumstances present at that particular moment of the procedure.

#### 2.4.2. Environmental Conditions

The ambient temperature in the operating theatre was 18 ± 0.5 °C, and the humidity was maintained at 45 ± 1%. Postoperative thermographic measurements were performed in the intensive care unit (ICU), where the ambient temperature was approximately 23 ± 0.5 °C, and the humidity was maintained at 45 ± 1%.

#### 2.4.3. Operating-Theatre Measurements

The patient was under general anaesthesia during the operative measurements. For facial thermographic imaging in the operating theatre, the monitored skin remained uncovered and, whenever clinically feasible, direct contact, pressure, rubbing, warming, cooling, and unnecessary manipulation of the monitored areas were avoided. Because intraoperative thermographic acquisitions were performed during active flap preparation, transfer, inset, and reconstruction, a formal 30 min thermal stabilisation period could not be applied before every intraoperative measurement. During the operative procedure, the flap was left undisturbed whenever possible, including avoidance of manipulation for at least 5 min before selected intraoperative image acquisitions.

During operating-theatre measurements, the imaging environment was monitored to ensure that no reflective elements or additional radiation sources were present in the background of the imaged areas, as these could affect the accuracy of the recorded temperature values.

The intraoperative component was included because thermography could be performed directly in the operating theatre without tissue contact or ionising radiation. In this case, intraoperative measurements provided descriptive information on surface-temperature patterns and asymmetry between the skin islands at the reconstructed operative stages. This operating-theatre applicability was the study’s main technical feature and distinguished the approach from purely postoperative flap monitoring.

Intraoperative thermographic observations were interpreted retrospectively in relation to the reconstructed operative stages and available vascular assessments, following completion of the blinded ROI delineation and temperature calculations. Neither ischaemia duration nor post-reperfusion rewarming rates were estimated.

#### 2.4.4. Postoperative ICU Measurements

After surgery, thermographic monitoring continued in the ICU. Before each postoperative thermographic examination, the monitored facial areas were left undisturbed for at least 30 min to allow facial skin acclimatisation and thermal stabilisation under the ICU environmental conditions.

#### 2.4.5. Interpretation of Intraoperative and Postoperative Measurements

No normalisation was performed between the operating-theatre and ICU measurements. Because ambient temperature, tissue exposure, handling, and thermal stabilisation conditions differed between these settings, intraoperative and postoperative observations were interpreted separately. Mean surface temperatures were retained as descriptive values within each phase and were not treated as directly comparable between phases or interpreted as a single continuous physiological trajectory. Temperature differences across the transition from the operating theatre to the ICU were therefore not attributed solely to changes in flap perfusion.

Inter-island comparisons refer to the same nominal time point and do not imply identical local exposure, head position, camera angle, or dressing conditions on both sides. All quantitative thermographic analyses were performed retrospectively and did not inform intraoperative or postoperative clinical decision-making.

### 2.5. ROI Analysis

Thermographic images were acquired by a person who did not participate in the surgical procedure. Quantitative thermographic analysis was performed retrospectively using ThermaCAM Researcher Pro 2.10 software. Regions of interest (ROIs), defined as areas of the thermogram selected for detailed analysis, were manually delineated at all analysed time points by the same experienced researcher to maintain consistency in ROI selection.

For serial whole-island analysis, the ROI was manually delineated along the visible cutaneous boundary of each skin island. The contour was placed as close as technically possible to the suture line, including the skin immediately adjacent to the narrow dressing covering the suture, while restricting the ROI to directly visible flap skin. Non-cutaneous tissue was excluded. Sutures and areas containing adherent dried blood were also excluded from the ROI.

Wound margins were included only up to the visible cutaneous edge. Areas that were obscured by dressings, surgical instruments, or other objects were excluded, and no attempt was made to extrapolate the ROI through non-visible regions. Areas containing visible blood or moisture were excluded when these prevented confident identification of the underlying skin surface. No interpolation of excluded pixels was performed. Consequently, each ROI represented the largest clearly visible continuous skin-covered portion of the respective skin island that could be delineated without including non-cutaneous tissue or foreign/material-related structures.

Prior to quantitative analysis, thermographic images were anonymised and assigned coded image-identification numbers. The analyst had no access to patient-identifying information, medical records, clinical examination findings, Doppler ultrasonography or computed tomography angiography (CTA) results, treatment decisions, or the final clinical outcomes of the monitored skin islands.

Acquisition time points and right/left side designation were also masked during ROI delineation and temperature calculations. The coded thermograms did not contain information identifying the acquisition time point, and the chronological timing of each image was reconstructed only after completion of the quantitative analysis by linking the image-identification numbers to the acquisition records. Explicit right/left labels were not provided to the analyst. During ROI delineation, the researcher relied only on the anatomical appearance visible on the thermogram, including the general orientation of the patient’s head. Masking, therefore, concerned explicit labels; anatomical cues could still permit inference of side.

Image selection was based on technical quality rather than random sampling because not all acquired images were technically suitable for reliable quantitative assessment. Images with insufficient sharpness or with skin-island boundaries that could not be delineated confidently were excluded from quantitative analysis. Image inclusion was therefore based on technical image quality, primarily adequate sharpness and clear visualisation of the skin-island surface and its boundaries. When more than one acquisition was available for a given assessment episode, the technically best-quality image suitable for reproducible ROI delineation was selected.

Image-quality selection was performed independently of the subsequent blinded quantitative assessment. Thus, ROI delineation and temperature calculations were carried out on anonymised, coded images without disclosure of clinical information, acquisition time point, or side. The retrospective thermographic analysis did not inform clinical decision-making.

For comparisons across the measurement series, the main ROI corresponded to the visible surface of each skin island. At 0 h, with the vascular pedicle intact and before division of the flap into two skin islands, two separate ROIs were delineated on the intact flap. Their size and location corresponded as closely as possible to the areas subsequently analysed on the individual left and right skin islands. Separate central, distant, and reference ROIs were neither predefined in the available data nor introduced retrospectively. Intra- and inter-operator repeatability of ROI delineation was not formally assessed.

For each ROI, the mean temperature was calculated and used as the primary descriptive thermal parameter for subsequent comparison with assessments of flap and skin-island perfusion and viability. The standard deviation (SD) of the temperature values of all pixels contained within each ROI was also calculated to characterise spatial temperature heterogeneity within the analysed skin surface. Because these pixels were spatially related observations derived from a single thermographic image, within-ROI SD was treated solely as a descriptive measure of spatial temperature distribution. It does not represent repeated-measurement variability, technical measurement error, uncertainty of the ROI mean, camera precision, or measurement repeatability. Technical repeatability was not assessed because replicate thermographic acquisitions under identical conditions were not performed for this purpose.

Inter-island thermal asymmetry was expressed as the absolute temperature difference (|ΔT|), calculated as the absolute difference between the mean temperatures of the right and left skin-island ROIs. Thus, |ΔT| represented the magnitude of thermal asymmetry between the two regions, irrespective of which side exhibited the higher temperature.

In addition to the prespecified whole-island analysis, retrospective visual review of the postoperative thermograms identified a focal area of locally increased surface temperature within the viable left skin island, hereafter referred to as a “hot spot”. This observation was not a prespecified quantitative endpoint. The hot spot was identified qualitatively as a visually distinct local area of higher surface temperature relative to the immediately surrounding skin on the same thermogram.

No predefined temperature-difference threshold fixed hot-spot ROI geometry, or standardised surrounding reference ROI was used. Accordingly, the hot spot was not analysed as a quantitative diagnostic parameter, and no numerical cutoff or diagnostic threshold was derived. Any subsequent comparison between the location of the visually identified hot spot and Doppler findings was performed after the thermographic review and was not used to define the hot spot during the blinded thermographic analysis. The observation was therefore considered qualitative, exploratory, and hypothesis-generating rather than a validated marker of flap viability, perforator function, or local perfusion.

### 2.6. Standard Flap Monitoring

During the intraoperative phase, Doppler ultrasonography was performed before flap division into two skin islands (0 h). Doppler ultrasonography was not performed at 3 h and 4 h (Table 1). Postoperative Doppler examinations corresponded to the thermographic observation time points of 20 h, 28 h, 44 h, 49 h and 68 h, permitting retrospective comparison of thermographic and vascular findings within the respective assessment episodes. These labels do not imply simultaneous examinations; at 49 h, thermography was performed after the clinical, Doppler and CTA results were known. The results of all Doppler examinations performed at the analysed time points (5 h, 7 h, 20 h, 28 h, 44 h, 49 h and 68 h) are presented together with the thermographic observations. At 0 h, an audible Doppler signal was present in the main vascular pedicle and in both supplying perforators. From 5 to 44 h, handheld Doppler examination consistently demonstrated audible flow in both skin islands. At 49 h, no audible Doppler signal was detected from the right skin island, whereas the signal remained detectable in the left skin island. At 68 h, only the remaining left skin island was assessed, and an audible Doppler signal was still present.

### 2.7. Outcomes and Interpretation Framework

The primary feasibility outcome was the ability to obtain interpretable serial thermograms of both skin islands intraoperatively and postoperatively without tissue contact, alongside standard monitoring. Feasibility was limited to obtaining interpretable images; acquisition speed, completion rate, and absence of disruption were not quantified in this analysis. Descriptive thermographic variables included mean surface temperature within the manually delineated ROIs, inter-island thermal asymmetry, temporal temperature trends within each phase, focal areas of increased temperature, and abrupt thermal decreases. Clinical outcomes of interest were the need for surgical revision, partial skin-island necrosis, and preservation or loss of the monitored tissue. Thermographic findings were retrospectively compared with documented Doppler ultrasonography and CTA findings, bleeding response to puncture, skin colour, and the clinical assessment of skin-island viability.

### 2.8. Statistical Analysis

Because the analysis included one patient and two non-independent skin islands, no inferential statistical testing was performed. Mixed-effects models, receiver operating characteristic (ROC) analysis, sensitivity/specificity estimates, and intraclass correlation coefficients (ICC) would not be statistically valid for the present dataset and were therefore not calculated.

Temperature values, inter-island differences, and temporal trends are reported descriptively and separately for the intraoperative and postoperative phases. Temperature values, inter-island differences, and temporal trends were analysed descriptively. Within-ROI SD was calculated from the pixel temperature values contained within a single ROI and represents spatial thermal heterogeneity only. Pixel values within an ROI were not treated as independent observations or technical replicates. Accordingly, within-ROI SD does not estimate uncertainty of the mean, measurement error, camera precision, or technical repeatability. Technical repeatability was not assessed in the present study.

### 2.9. Ethics

The scientific analysis was performed in accordance with the Declaration of Helsinki within the observational project entitled “Ocena przydatności termografii w śródoperacyjnym i pooperacyjnym monitorowaniu perfuzji płatów oraz funkcji zespoleń mikrochirurgicznych w chirurgii rekonstrukcyjnej”. Ethical approval for the observational analysis was granted by the Ethics Team of the Wojewódzki Specjalistyczny Szpital Dziecięcy w Olsztynie (Certificate No. 19 ZE/2026/WSSD; approval date: 14 July 2026). The index operation was performed on 19 August 2025, and thermographic images were acquired as an additional procedure during the operative and postoperative course. The retrospective quantitative analysis of the recorded thermograms was performed following the approval granted on 14 July 2026. Based on the written informed consent of the patient’s legal guardian, permission was obtained to perform thermographic examinations and to use anonymised clinical data and illustrative materials for scientific publication purposes (approval—18 August 2025).

## 3. Results

The thermographic findings presented below were obtained through retrospective analysis of images acquired before, during, and after surgery. Thermography did not influence clinical decision-making, including the decision to reoperate, which was based on clinical examination, Doppler ultrasonography, and computed tomography angiography (CTA).

**Figure 1:**
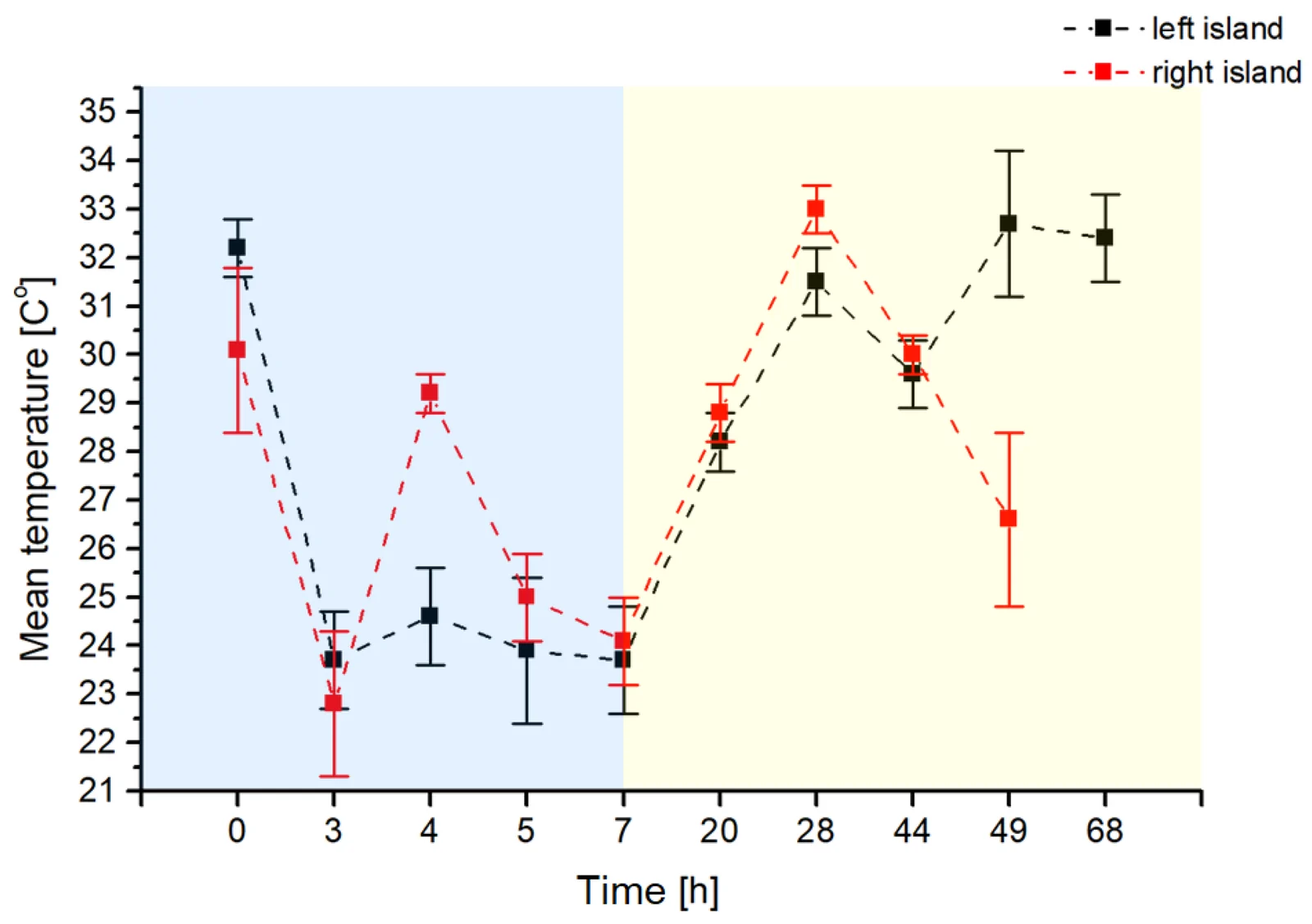
Mean temperature values calculated in the designated regions of interest (ROI) on the surface of the left and right skin islands, during surgery–intraoperative time 0–7 h (area of the chart marked in blue) and during postoperative monitoring-time 20–68 h (area of the chart marked in yellow). No normalisation was performed between the operating theatre and the ICU measurements. Absolute temperatures are descriptive within each phase and should not be directly compared between phases or interpreted as a single continuous physiological trajectory. Temperature differences across the transition between settings should not be attributed solely to changes in flap perfusion. Time 0 h was defined as the first thermographic measurement performed immediately after flap dissection and before division of the flap into two skin islands. At this time point, two separate ROIs were delineated on the intact flap, with dimensions corresponding to the ROIs subsequently analysed on the individual left and right skin islands. Within-ROI SD values are provided separately in Table 2 and Table 3 and describe spatial temperature heterogeneity among pixels within an individual ROI; they do not represent repeated-measurement variability, uncertainty of the mean, camera precision, or technical repeatability. Technical repeatability was not assessed.

The analysed series contained 10 flap-monitoring time points: 0, 3, 4, 5, 7, 20, 28, 44, 49, and 68 h after flap dissection. Both skin islands and their prospective regions were assessed at the first nine time points; only the remaining left island was assessed at 68 h. The numerical temperatures and inter-island differences presented below were derived retrospectively from these recorded images. In Figure 1, the intraoperative measurements (0–7 h, blue background) and postoperative ICU measurements (20–68 h, yellow background) are displayed as separate phases. Absolute temperatures are presented descriptively within each phase, not as a single continuous physiological trajectory across settings.

The Doppler findings at the intraoperative assessment points were as follows: at 0 h, an audible Doppler signal was detected in the primary vascular pedicle and both perforators supplying the region. From 5 to 44 h, audible blood flow was demonstrated in both skin islands. At 49 h, the Doppler signal was absent from the right skin island; however, it persisted on the left. By 68 h, only the left skin island was assessed, and an audible Doppler signal continued to be observed there.

**Figure 2:**
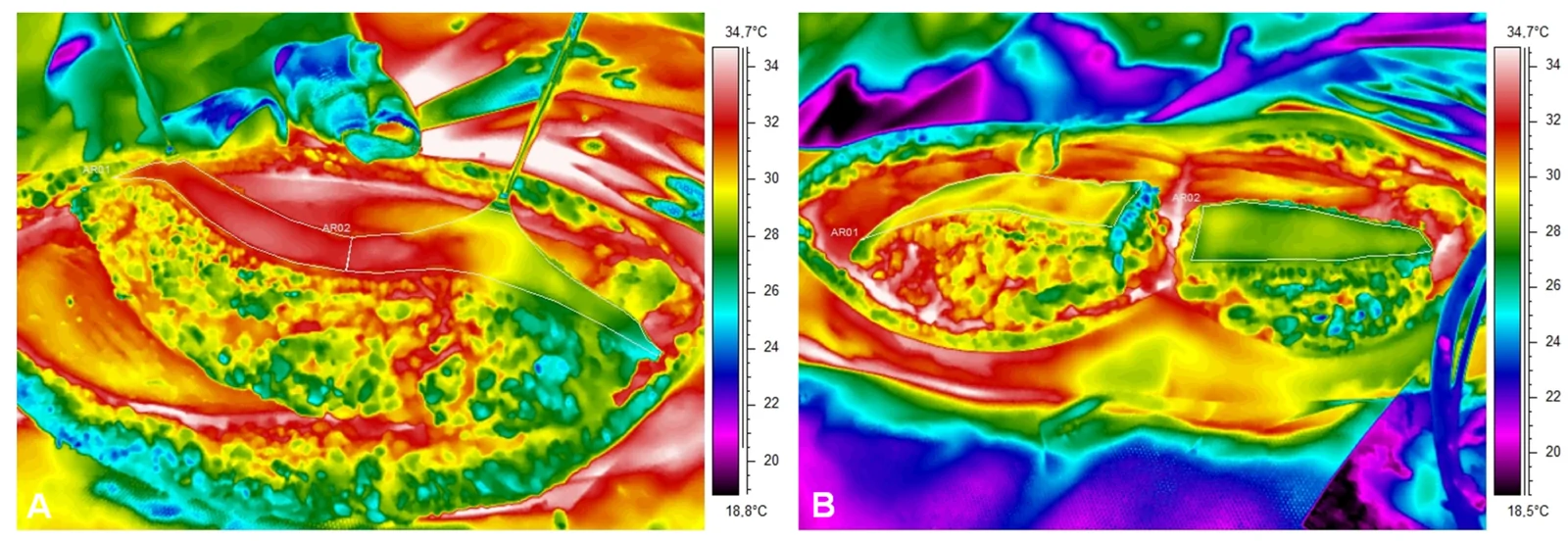
Thermogram of the flap (**A**) immediately after dissection and (**B**) after dividing it into two skin islands.

Before dividing the flap into two skin islands, a thermographic image was taken (Figure 2A). Thermal analysis of the surface of the flap before division showed that the part later designated as the right skin island had a temperature over 2 °C lower than the segment marked as the left skin island (Figure 1—time 0 h). After dividing the flap into two skin islands, a second thermographic image was taken (Figure 2B). Thermogram analysis confirmed that the island intended for the reconstruction of the right cheek exhibited a lower temperature compared to the island prepared on the left side of the face. A marked decrease in the mean surface temperature of both skin islands was observed in comparison to the average temperature values recorded in the corresponding areas immediately after flap dissection. This phenomenon occurred despite maintaining heating conditions, including contact with surrounding tissues and the use of dressings with warmed saline. The difference in mean temperatures between the left and right islands was 0.9 °C (Figure 1—time 3 h).

**Figure 3:**
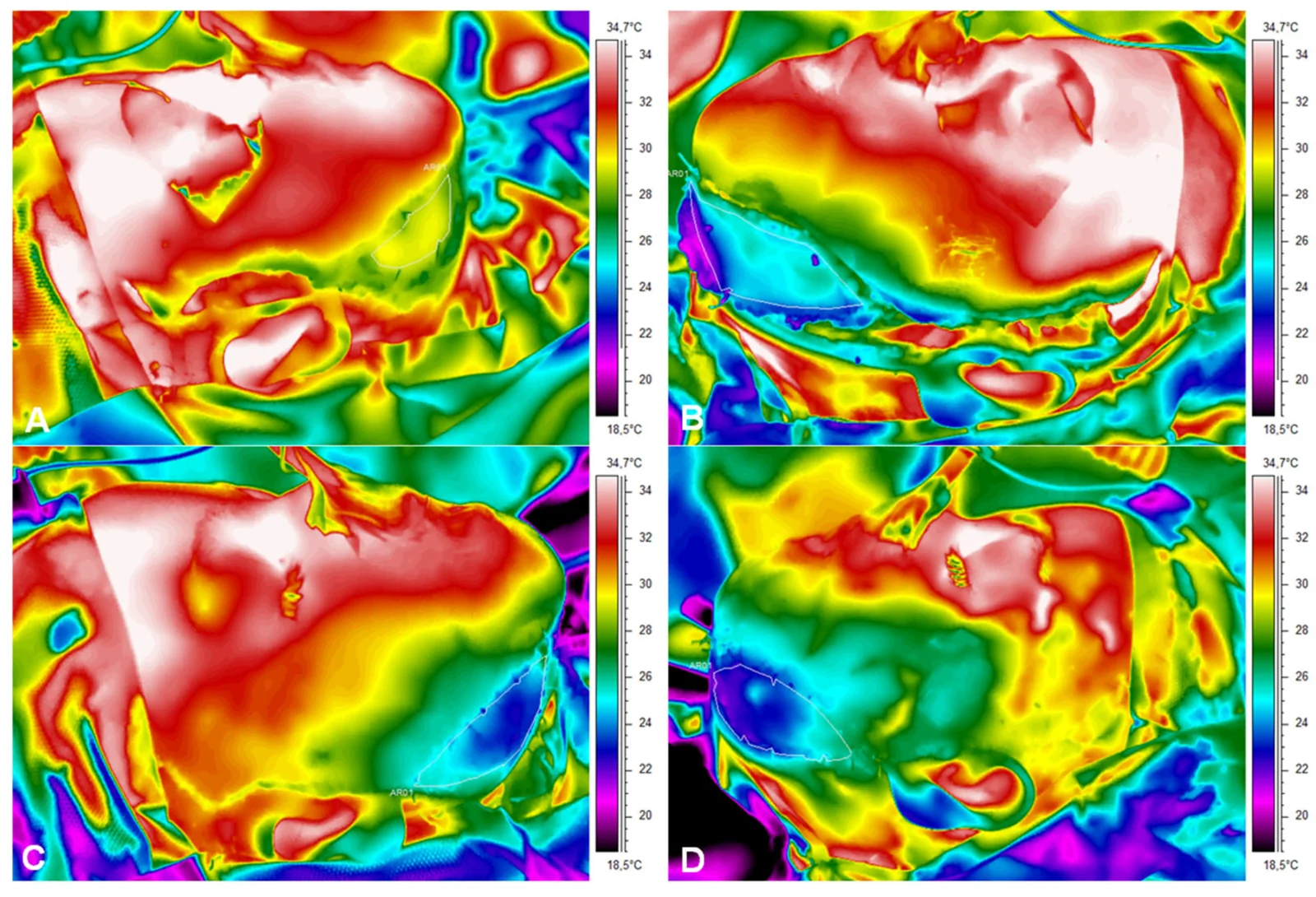
Thermograms of (**A**,**C**) the right and (**B**,**D**) left skin island recorded (**A**,**B**) intraoperatively 4 h and (**C**,**D**) 7 h after flap dissection.

The procedure required the involvement of a multidisciplinary team and included numerous surgical and microsurgical stages, lasting a total of approximately 8 h. For this reason, the surface temperature of the skin islands was serially monitored throughout the operation. During the procedure, marked fluctuations in the temperature of the skin islands were observed, particularly noticeable oscillations on the right island, whose average surface temperature showed greater variability. The left island, on the other hand, had a more stable thermal profile (Figure 1—times 4 and 5 h). Subsequent thermograms (Figure 3) were recorded during the operation, including during the fitting phase and the gradual suturing of the islands. Figure 3 presents the thermal parameters obtained at that time for the right and left islands, until their complete placement. It was also noted that after the final implantation, the right island reached a slightly higher temperature than the left (0.4 °C, Figure 1—time 7 h), despite the left island having a higher thermal value immediately after preparation from the thigh (>2 °C, Figure 1—time 0).

The corresponding mean surface temperatures of the left and right skin island ROIs, together with the within-ROI SD values, absolute inter-island temperature differences (|ΔT|), and the associated operative stages, are summarised in Table 2.

**Figure 4:**
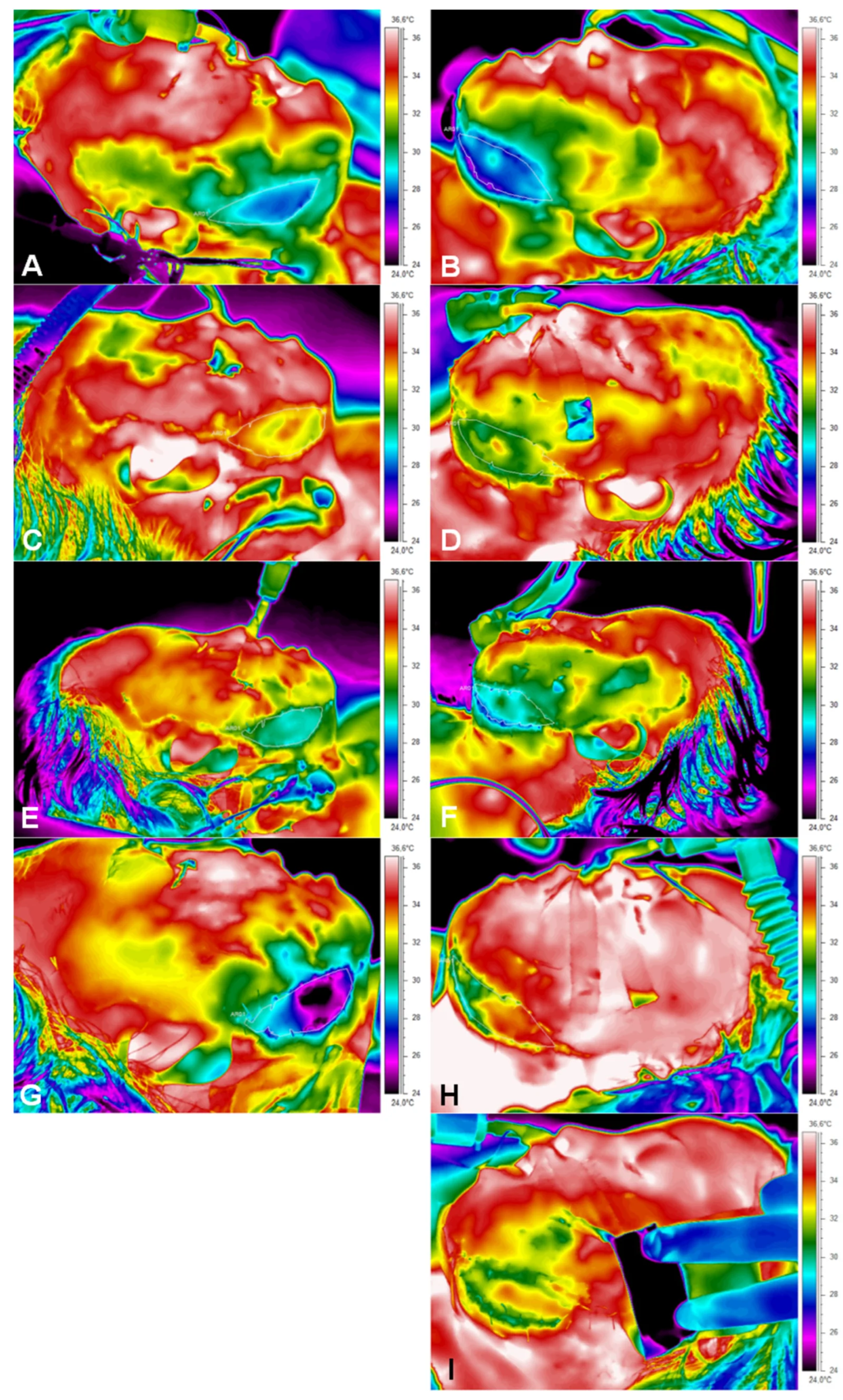
Thermograms of (**A**,**C**,**E**,**G**) the right and (**B**,**D**,**F**,**H**,**I**) left skin island recorded in the intensive care unit (**A**,**B**) 20 h, (**C**,**D**) 28 h, (**E**,**F**) 44 h, (**G**,**H**) 49 h, (**I**) 68 h after flap dissection. A focal area of relatively increased surface temperature (“hot spot”) was identified visually within the viable left skin island. The hot spot was identified retrospectively and qualitatively, without a predefined temperature threshold or standardised surrounding reference ROI, and should be interpreted as an exploratory observation only.

The patient was transferred to the intensive care unit and subjected to analgesic sedation, which involved the simultaneous administration of painkillers and sedatives. As a result, the patient remained conscious from the anaesthesia but in a controlled, shallow sleep state, effectively reducing pain and stress after the extensive surgery. The temperature of the transplanted flap was serially monitored postoperatively at the predefined time points (Figure 4, Table 3).

Postoperative monitoring followed the standard clinical protocol. Dressings were changed daily, and patency and functional status of the vascular anastomosis were assessed using Doppler ultrasonography. The postoperative Doppler assessments corresponded to the 20 h, 28 h, 44 h, 49 h and 68 h thermographic observation time points. These examinations were not necessarily simultaneous; in the 49 h episode, clinical assessment, Doppler ultrasonography and CTA preceded thermographic acquisition. Thermography was performed as an additional procedure to compare its findings with those of standard methods and was not used to guide management.

On the first day after the operation (Figure 4A,B, Figure 1—time 20 h), the temperature of the right island was about 0.6 °C higher than that of the left. This trend persisted up to 28 h after flap dissection, when the temperature of the right island was 1.5 °C higher than that of the left island. On the second day after the operation (Figure 4E,F, Figure 1—time 44), the temperature difference between the islands decreased markedly and was only 0.4 °C. In the following hours (Figure 4G,H, Figure 1—time 49 h), the left island exhibited a clearly higher temperature than the right island. The temperature difference between the islands exceeded 6 °C, and the average temperature of the right island was only 26.6 °C.

**Figure 5:**
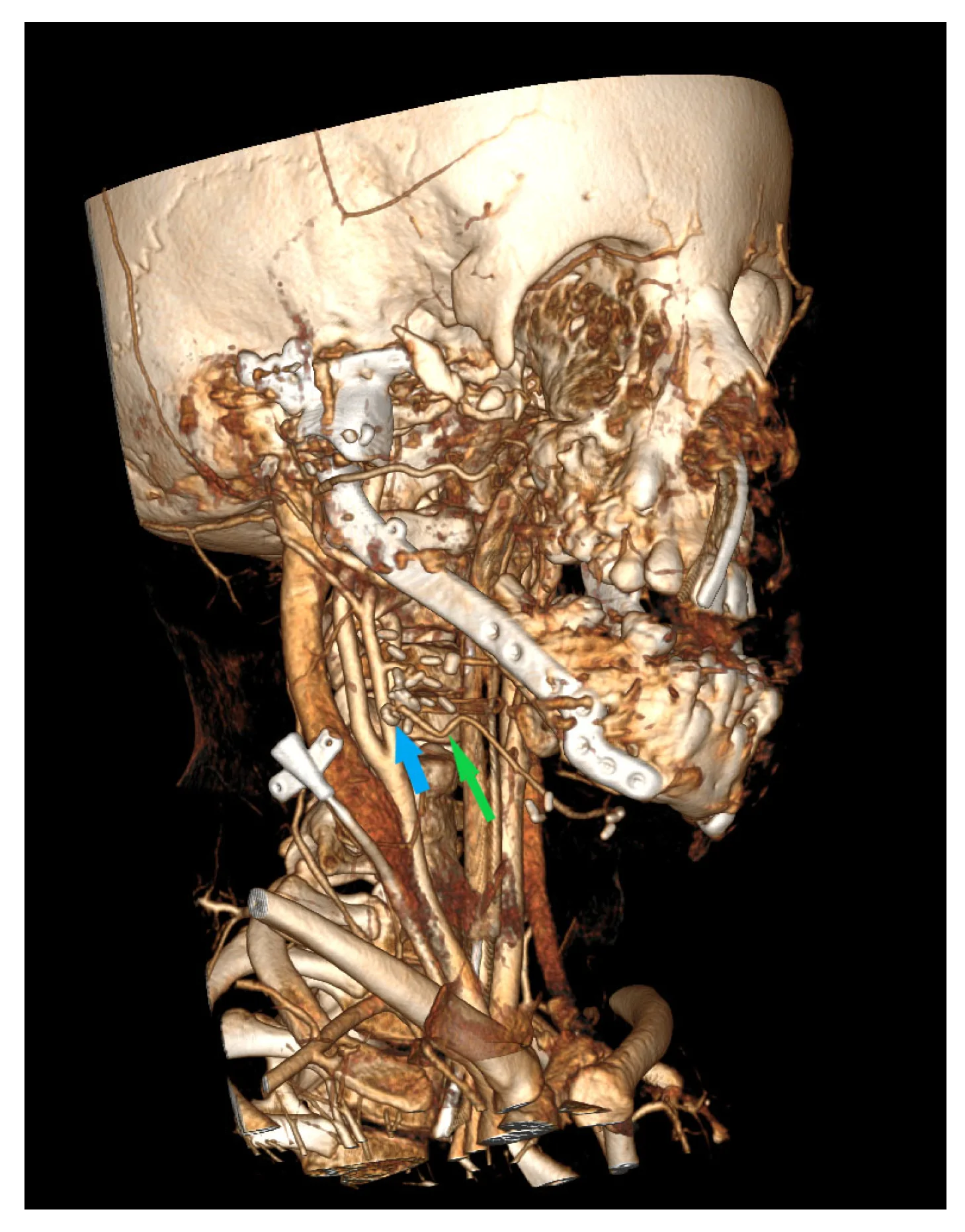
Three-dimensional (3D) reconstruction of bone structures, implants, and vascular structures obtained from computed tomography angiography (CTA). The CTA analysis focused on the vascular supply of the right skin island, including assessment of the expected perforator course. Blue arrow points to the coupler on the arterial anastomosis between the right facial artery and the artery of the vascular pedicle of the ALT flap; green arrow points to the visible perforator to the left skin island. The perforator to the right skin island is not visible; this finding is concordant with the absent audible signal from the right-perforator flow on examination with blind Doppler.

During the clinical episode corresponding to the 49 h thermographic observation, pallor of the right skin island was the first finding to raise concern. Examination with a blind Doppler probe was then performed, and no audible signal was obtained from the right perforator. CTA was performed next and did not visualise that perforator (Figure 5). Absence of bleeding after puncture was also recorded during the clinical assessment. Only after the results of these standard assessments had been communicated were the 49 h thermograms acquired, with the intention of assessing concordance with the already established clinical and vascular findings. The decision to reoperate and remove the non-viable right skin island was based on the clinical, Doppler and CTA findings, not on thermography. During the reoperation, it was found that thrombosis was limited to the perforator to the right skin island close to the main pedicle; salvage was not attempted due to advanced necrotic changes in the right skin island, especially in its de-epithelialized part, visible during reoperation. Anastomosis of the main pedicle to the facial vessels was examined during revision and was fully functional. After reoperation and removal of the necrotic skin from the island, a repeat thermographic examination of the patient was performed, focusing on the remaining, functioning left island (Figure 4I).

The temperature values reported for the 49 h time point were calculated retrospectively from the images acquired during that episode, following ethical approval granted on 14 July 2026. They were not available as results of this analysis during treatment. Thermography did not identify the problem before the standard methods or independently alter the decision to reoperate; the observed agreement represents retrospective descriptive concordance (Table 4). The sequence is reported above, but exact clock times and intervals between the individual assessments are not specified in this report.

The thermographic values in Table 4 were calculated retrospectively and are presented alongside the clinical and vascular findings recorded during treatment. The inter-island temperature difference was not used as a threshold or criterion for reoperation. The table, therefore, illustrates retrospective descriptive concordance, not a thermography-guided decision pathway.

On retrospective review of the postoperative thermograms, a focal area of locally increased surface temperature was visually identified within the viable left skin island (Figure 4B,D,F,H,I). The same general region showed preserved vascular flow on subsequent comparison with the available Doppler findings. However, the Doppler findings were not used to define or localise the hot spot during the blinded thermographic analysis.

Because the hot spot was identified retrospectively without a prespecified temperature threshold or a standardised surrounding reference ROI, it is reported only as a qualitative and exploratory thermal pattern. It should not be interpreted as a validated diagnostic marker of flap viability, perforator function, or local tissue perfusion. Moreover, the thermographic findings therefore reflect a surface-temperature pattern associated with local perfusion compromise and should not be interpreted as evidence of failure of the entire vascular pedicle or the principal microvascular anastomoses.

The final clinical outcome of the described case was assessed at one-year follow-up. The remaining skin island and all other surgical sites were fully healed. The patient is still undergoing physiotherapy to achieve full function of the TMJ prosthesis. No subsequent reconstructive intervention was required.

## 4. Discussion

The present single-patient retrospective technical feasibility analysis yielded three main findings. First, serial infrared thermography was technically feasible for non-contact intraoperative and postoperative monitoring of the transplanted skin islands. Second, thermographic observations showed descriptive concordance with the documented clinical and vascular findings, including intraoperative inter-island temperature asymmetry, postoperative temperature divergence, and a marked temperature decrease in the right skin island at 49 h. Third, a focal area of relatively increased surface temperature was observed retrospectively within the viable skin island. Because this finding was identified qualitatively without a prespecified temperature threshold or standardised surrounding reference ROI, it should be regarded only as an exploratory, hypothesis-generating observation and not as a validated marker of flap viability or perforator-level perfusion. Importantly, thermography did not guide the diagnosis of vascular compromise or the decision to reoperate. These findings support the feasibility of serial thermal documentation as an adjunct to standard flap monitoring, while the single-case retrospective design does not allow conclusions regarding earlier detection, diagnostic accuracy, or clinically actionable temperature thresholds.

Despite significant progress in reconstructive surgery, the treatment of complications related to flap vascularisation remains a matter fraught with substantial knowledge gaps and insufficiently explored in the literature [14,15]. The analysis involved a detailed assessment of the relationship between the temperature of the free flap taken from the patient’s thigh used for mandibular reconstruction and its postoperative function. According to available literature, paediatric patients under the age of 10 may be at increased risk of a lower success rate with this type of reconstructive procedure [11,14]. Among the potential mechanisms underlying this phenomenon are the smaller blood vessel diameter in younger patients, a tendency toward arterial constriction, and greater technical difficulty in performing vascular anastomosis on smaller anatomical structures.

At the same time, the literature contains numerous reports that do not confirm a significant correlation between the patient’s age and the frequency of complete flap loss, contradicting the above observations [14,16]. The coexistence of these differing views suggests that although the patient’s age is undoubtedly an important clinical factor, it does not necessarily have to be a critical factor determining skin island survival. In the present case, vascular compromise resulted in necrosis and surgical removal of the right skin island, whereas the remaining left skin island and the transferred flap tissue were preserved. Therefore, this event represents partial skin-island loss rather than total free-flap failure. The thermographic pattern should not be interpreted as direct evidence of failure of the main vascular pedicle or microvascular anastomoses.

As a result, a comprehensive assessment of the entire therapeutic process becomes particularly important, including the stage of tissue harvesting, the conditions of its storage until implantation, as well as close monitoring of its condition during the postoperative period.

Postoperative monitoring of free flaps in current medical practice can be carried out using a range of advanced diagnostic methods, including near-infrared spectroscopy (NIRS), indocyanine green fluorescence angiography (ICG-FA), fluorometric techniques, tissue oximetry, and laser imaging. Simultaneously, in clinical practice, traditional and relatively simple methods are still employed, such as tissue puncture tests, blind Doppler examinations, or clinical assessment of the appearance and colour of the graft [3,4,17,18,19,20]. A key element of diagnostic evaluation remains the analysis of the degree and quality of perfusion of the transplanted flap.

Despite the wide range of available techniques, there remains a demand for a non-invasive solution that enables real-time data acquisition and early detection—already during flap harvesting—of perfusion disturbances and the risk of reconstruction failure. In this context, infrared thermographic imaging appears to be a promising investigational adjunct, enabling rapid, contactless, and straightforward assessment of the surface temperature of both transplanted tissues and adjacent structures. The rapid development of this technology offers the possibility of surface-temperature assessment relevant to the evaluation of blood supply without the need for direct contact with the tissue [3,21].

Available studies, including animal models, in vitro analyses of human tissues, and in vivo observations in patients after reconstructive procedures, have explored the usefulness of thermography as a method for localising vessels within transplanted flaps. Furthermore, this technique has been investigated for the delineation of ischemic and necrotic areas [2]. An additional noteworthy point is the increasing number of reports concerning the use of low-cost thermal imaging cameras integrated with smartphones, both in postoperative monitoring of flaps and in identifying perforators during the planning stage of reconstructive surgeries [3,4,22]. Current research results indicate that infrared thermography can be applied at all stages of tissue transfer procedures, serving as a valuable complement to standard diagnostic methods [3,21].

Phillips et al. [4] reported lower temperatures in DIEAP flaps with either arterial insufficiency or venous congestion, with rewarming after correction of the flow disturbance. The lower temperature of the non-viable right island in the present case is descriptively consistent with those observations. However, differences in flap anatomy, acquisition conditions, and operative timing preclude transferring temperature values or diagnostic interpretations directly to this paediatric ALT reconstruction.

Recent clinical studies provide additional context for the postoperative temperature divergence observed in the present case. Chava et al. prospectively evaluated infrared thermography in 20 patients undergoing head and neck microvascular reconstruction. They reported concordance between thermographic hotspots and perforator localisation in 93% of the cases assessed preoperatively, demonstrated intraoperative flap rewarming in 19 of 20 cases, and observed postoperative thermographic abnormalities in three compromised flaps before corresponding abnormalities became apparent on clinical monitoring [23]. Importantly, however, decisions regarding re-exploration in that study were based on clinical assessment rather than thermography.

Singla et al. subsequently evaluated 41 free flaps, including five with vascular compromise, using serial infrared thermography together with conventional clinical monitoring. Their analysis was based primarily on the temperature difference between the flap and adjacent reference skin. The authors reported substantially greater temperature differences in compromised than in viable flaps and proposed a ΔT cutoff of 2 °C, with a sensitivity of 88.6% and specificity of 98.9% [24]. These findings support the concept that relative rather than isolated absolute surface temperature may be informative. However, they cannot be directly transferred to the present case. Our analysis involved a single patient; no predefined adjacent reference-tissue ROI was available, operating-theatre and ICU conditions differed, and the marked 49 h thermal asymmetry was analysed retrospectively after the clinical, Doppler, and CTA findings had already been established. Consequently, the >6 °C inter-island difference observed here should be regarded as a descriptive finding rather than a diagnostic threshold or evidence of earlier detection.

The exploratory focal hot spot observed in the viable left skin island should likewise be interpreted cautiously in the context of recent perforator-mapping studies. Saleem et al. studied 28 patients undergoing head and neck free-flap reconstruction, including 20 ALT flaps, using smartphone-based dynamic thermography after a controlled cooling challenge. Thermographically identified hot spots generally lay within 0–2 cm of surgically identified perforators, although the comparisons did not reach statistical significance [25]. Similarly, Meier et al. evaluated DIRT in 20 patients undergoing ALT flap reconstruction and found high concordance between projected thermal hot spots and handheld Doppler signals, while correspondence with the exact intraoperative perforator location depended on the permitted spatial distance [26]. These studies support a potential spatial relationship between focal surface-temperature patterns and underlying perforator territories, but they used controlled dynamic cooling–rewarming protocols specifically designed for perforator localisation. In contrast, the present hot spot was identified retrospectively on serial static thermograms without a controlled thermal challenge, predefined thermal threshold, or standardised surrounding reference ROI. It therefore remains a qualitative, hypothesis-generating observation and should not be interpreted as a validated marker of perforator-level perfusion.

This distinction is also consistent with the recent systematic review by Clarys et al., which included 31 studies of dynamic infrared thermography in free-flap surgery and specifically required a dynamic cold challenge, excluding studies based solely on static thermography. The review reported promising performance of DIRT for perforator detection but also emphasised heterogeneity of protocols and the need for standardisation and larger prospective studies [1]. Accordingly, diagnostic performance estimates derived from DIRT should not be extrapolated to the serial static protocol used in the present study. Our observations instead demonstrate the feasibility of acquiring serial surface-temperature information during clinically defined operative and postoperative stages, while the diagnostic significance of such static thermal patterns requires prospective validation.

The intraoperative contribution of this case is the feasibility of serial surface-temperature documentation across clinically defined stages of reconstruction. Linking the observations to pedicle division, completion of vascular anastomoses, reperfusion observed during inset, and assessment after final inset provides a more specific clinical context than reference to flap dissection alone.

The reconstructed operative timeline does not resolve the exact duration of flap ischemia. Pedicle division corresponds to the 3 h time point, and both vascular anastomoses were complete at the 4 h assessment, but separate arterial and venous completion times and the exact time of vascular clamp release and restoration of blood flow were not available for this analysis. Gradual reperfusion observed at 5 h is a qualitative operative observation, not a quantified delay in reperfusion. No exact ischemia duration or standardised interval after reperfusion was therefore calculated. The intraoperative temperatures should be interpreted as descriptive observations at surgical stages, not as a time-aligned ischemia–reperfusion curve or evidence of a causal relationship between temperature change and vascular function.

The acquisition used a camera with high thermal sensitivity and spatial resolution, along with regular calibration and blackbody validation. These technical parameters and quality-control procedures do not, by themselves, establish biological validity, diagnostic accuracy, or reproducibility of the clinical interpretation.

A significant limitation of the discussed method is that the analysis concerns only the surface temperature of tissues, which remains susceptible to numerous external factors. The resulting thermal image is affected by environmental conditions, such as ambient temperature and humidity, as well as the use of irrigation fluids in the surgical field and the presence of equipment routinely used in the operating room. Any element that generates a heating or cooling effect in direct contact with the monitored area can distort the recorded thermal data. Clinical experiences indicate that a particularly important role in this regard is played by interference from warming blankets, surgical sponges, irrigation systems, preparations used for surgical field preparation, and ultrasonographic gels, all of which significantly modify the distribution of surface temperature.

Another important limitation is the single-participant design. Although the study contains repeated thermographic measurements and therefore provides original methodological data, the observed temperature differences cannot be generalised without prospective validation. The descriptive ΔT-based interpretation should be treated as hypothesis-generating and should be tested in larger cohorts, ideally with standardised acquisition, predefined ROI selection and comparison with Doppler, indocyanine green angiography, tissue oximetry, and clinical outcomes.

Several additional limitations are relevant. First, ambient temperature differed between the operating theatre (18 °C ± 0.5 °C) and the ICU (approximately 23 °C ± 0.5 °C), as did tissue exposure, handling, and thermal stabilisation conditions. No normalisation was performed between these settings. Absolute intraoperative and postoperative temperatures were therefore interpreted separately as descriptive data within each phase, not as directly comparable values or a single continuous physiological trajectory. Temperature differences across the transition between settings should not be attributed solely to changes in flap perfusion. Second, central, distal, and external reference-tissue ROIs were not predefined, and the ROIs were manually delineated by one experienced researcher, precluding formal assessment of inter- or intra-rater reproducibility. Third, systemic temperature, hemodynamic variables, and exposure to vasoactive medications were not incorporated into the present analysis and may influence surface temperature. Finally, the available dataset does not support ROC analysis, sensitivity/specificity estimation, or a clinically actionable ΔT threshold.

Several potential sources of bias should be considered when interpreting the present findings. First, the single-patient and unusual case design introduces selection bias and substantially limits the generalisability of the observations. Second, the intraoperative measurements were obtained under the actual conditions of the surgical procedure. Although camera position, imaging distance, exposure, lighting, and skin-surface exposure were standardised whenever feasible, surgical manipulation, tissue handling, local exposure, and other procedure-related conditions could not be fully controlled or standardised. Similarly, no formal thermal stabilisation period could be applied before every intraoperative acquisition. These factors may have contributed to variability in the recorded surface temperatures. Differences in ambient conditions, tissue exposure, handling, and thermal stabilisation between the operating theatre and ICU were addressed by analysing the two phases separately; however, no normalisation between these settings was performed.

Third, central, distal, and external reference-tissue ROIs were not predefined, and ROI delineation was performed manually by a single experienced researcher. Intra- and inter-operator repeatability was not formally assessed, introducing the possibility of measurement and operator-dependent bias. The analyst was blinded to patient-related clinical information, including clinical examination, Doppler ultrasonography, CTA findings, treatment decisions, and final clinical outcomes. Moreover, image laterality and acquisition time were also masked. The quantitative thermographic analysis was retrospective and did not influence clinical decision-making. Accordingly, the observed concordance between thermographic findings and standard clinical and vascular assessments should not be interpreted as evidence of earlier detection, diagnostic performance, or an effect on patient management. Technical repeatability was not assessed because repeated thermographic acquisitions under identical conditions were not obtained for repeatability analysis. The reported within-ROI SD characterises spatial temperature heterogeneity within individual thermograms and should not be interpreted as a measure of measurement reproducibility or precision.

Furthermore, thermographic imaging provides information solely about the surface temperature of tissues, without enabling the assessment of either arterial inflow or venous outflow within the transplanted flap. As a result, based solely on thermal analysis, it is not possible to definitively determine the aetiology of microvascular disturbances. In this context, indocyanine green fluorescence angiography provides a broader range of diagnostic data, allowing real-time assessment of flap perfusion during both inflow and outflow phases. The anatomical and functional information obtained in this way is of significant importance when making decisions regarding potential surgical revision.

The transiently higher right-island temperature at 28 h cannot be attributed to inflammation, venous congestion, or arterial insufficiency from thermography alone. The mechanism of that temperature difference remains undetermined.

The present observations provide preliminary information relevant to future clinical translation. In this case, thermography was used as additional non-invasive, non-contact documentation alongside standard flap monitoring, enabling retrospective assessment of serial surface-temperature trends. Its practical feature was used already during surgery without tissue contact or additional ionising radiation. However, because the thermal image reflects surface temperature rather than direct arterial inflow or venous outflow, thermography should be integrated into a multimodal decision pathway rather than used as a standalone diagnostic tool. Future studies should investigate reproducible thermal thresholds, assess sensitivity and specificity, and determine whether intraoperative thermographic monitoring may provide clinically useful information for flap surveillance and subsequent management.

A subsequent prospective cohort study should predefine acquisition time points anchored to documented surgical events, including pedicle division, separate arterial and venous anastomosis completion, vascular clamp release and reperfusion, and final inset, together with ambient and patient-temperature controls, camera angle and distance, whole-island and central/distal/reference ROIs. Such a study should also incorporate predefined criteria for image interpretation and, where feasible, blinded assessment of thermograms. With an adequately sized cohort, repeated-measures mixed models could characterise thermal trajectories, ICC could quantify measurement reproducibility, and ROC analysis could explore whether any ΔT-based metric may have clinically useful discriminatory performance for outcomes such as surgical revision, partial skin-island necrosis, or total free-flap loss.

The practical contribution of this single case is the demonstration that serial infrared thermography can be performed intraoperatively and postoperatively as a non-contact adjunct to standard free-flap monitoring. In this reconstruction, thermography provided serial information on surface-temperature patterns that was retrospectively concordant with clinical and vascular findings, including a marked inter-island temperature difference at 49 h and a focal hot spot in the viable skin island. However, thermography did not guide clinical decision-making and did not demonstrate earlier detection or diagnostic accuracy. Therefore, its current clinical relevance in this setting lies in its feasibility as an additional source of non-invasive thermal information alongside established monitoring methods, rather than as a replacement for them.

## 5. Conclusions

In this single-patient retrospective technical feasibility analysis, serial infrared thermography was technically feasible for non-contact intraoperative and postoperative documentation of two skin islands and showed retrospective descriptive concordance with documented clinical and vascular assessments. These observations are limited to one reconstruction and do not establish earlier detection, diagnostic performance, a clinically actionable decision threshold, or superiority over standard flap monitoring. Further prospective studies are required to determine reproducibility and clinical utility. Thermography should therefore be regarded as an additional source of surface-temperature information alongside established monitoring methods, not as a replacement for them.

## Figures and Tables

**Table 1:** Perioperative clinical monitoring and additional infrared thermographic acquisition for the right and left skin islands. The investigations corresponding to the 49 h observation were sequential, as described in Section 3.

Phase	Time from Flap Dissection	Thermographic Context/Operative Stage	Doppler Ultrasonography	Clinical Assessment
Baseline (preoperative)	-	Baseline infrared thermographic imaging after facial skin acclimatisation.	Not performed.	Baseline clinical assessment before surgery.
Intraoperative	0 h	Flap dissection, prior to pedicle division.	Doppler ultrasonography was performed to assess vascular status during the reconstructive procedure. An audible Doppler signal was present in the main vascular pedicle and in both supplying perforators.	Intraoperative assessment of tissue appearance and perfusion.
Intraoperative	3 h	Division of the vascular pedicle and onset of flap ischemia.	Not performed.	Not performed.
Intraoperative	4 h	Arterial and venous microvascular anastomoses completed.	Not performed.	Clinical assessment, including the milking test and assessment of bleeding from flap margins.
Intraoperative	5 h	Initiation of flap inset, with gradual reperfusion observed.	Doppler ultrasonography of the vascular pedicle, anastomosis, and supplying perforators. An audible Doppler signal was present in both skin islands. *	Clinical assessment, including skin colour evaluation and assessment of bleeding from flap margins.
Intraoperative	7 h	Assessment of the flap following completion of the final inset.	Doppler ultrasonography of the vascular pedicle, anastomosis, and supplying perforators. An audible Doppler signal was present in both skin islands. *	Clinical assessment, including skin colour evaluation and puncture test, with assessment of bleeding response.
Postoperative	20 h	ICU surveillance	Doppler ultrasonography of the vascular pedicle, anastomosis, and supplying perforator. An audible Doppler signal was present in both skin islands. *	Clinical assessment, including skin colour evaluation and puncture test, with assessment of bleeding response.
Postoperative	28 h	ICU surveillance	Doppler ultrasonography of vascular flow. An audible Doppler signal was present in both skin islands. *	Clinical assessment, including skin colour evaluation and puncture test, with assessment of bleeding response.
Postoperative	44 h	ICU surveillance	Doppler ultrasonography of vascular flow. An audible Doppler signal was present in both skin islands. *	Clinical assessment, including skin colour evaluation and puncture test, with assessment of bleeding response.
Postoperative	49 h	Additional thermographic acquisition after the clinical, Doppler and CTA results were known.	Doppler ultrasonography after pallor was noted and before CTA. An audible Doppler signal was present only in the left skin island. *	Pallor was the first sign of concern; absence of bleeding after puncture was also documented.
Postoperative	68 h	Follow-up thermography of the remaining viable left skin island after removal of the non-viable right island	Doppler ultrasonography of vascular flow. An audible Doppler signal was present in the remaining left skin island. *	Clinical assessment, including skin colour evaluation and puncture test, with assessment of bleeding response.

* The available source documentation did not allow for separate, vessel-specific confirmation of the presence of a signal in the main vascular pedicle and in each of the supplying perforators. All time points are referenced to the initial flap thermogram (0 h), obtained during dissection before pedicle division; they do not denote elapsed time from ischemia onset, reperfusion, or the end of surgery.

**Table 2:** Intraoperative mean surface temperatures of the left and right skin island ROIs at the analysed operative time points. Values are presented as mean temperature with within-ROI standard deviation (SD), calculated from all pixel temperatures within a single ROI and reflecting spatial temperature heterogeneity only; it does not represent repeated-measurement variability, uncertainty of the mean, camera precision, or technical repeatability. |ΔT| denotes the absolute difference between the mean temperatures of the right and left ROIs. Technical repeatability was not assessed.

Figure	Time [h]	Mean Temperature of the Left Skin Island ± Within-ROI SD [°C]	Mean Temperature of the Right Skin Island ± Within-ROI SD [°C]	|ΔT| [°C] (Right–Left)	Stage of Operation
Figure 2A	0	32.2 ± 0.6	30.1 ± 1.7	2.1	Preparation of a flap with an intact vascular pedicle, before the division of the pedicle
Figure 2B	3	23.7 ± 1.0	22.8 ± 1.5	0.9	Division of the vascular pedicle and the onset of ischemia of the skin islands
Figure 3A,B	4	24.6 ± 1.0	29.2 ± 0.4	4.6	Microanastomoses of arterial and venous vessels were performed
Not presented	5	23.9 ± 1.5	25.0 ± 0.9	1.1	The initiation of the flap inset and gradual reperfusion was observed
Figure 3C,D	7	23.7 ± 1.1	24.1 ± 0.9	0.4	Assessment of the flap after the final implantation

**Table 3:** Postoperative mean surface temperatures of the left and right skin island ROIs during ICU monitoring. Values are presented as mean temperature with within-ROI standard deviation (SD), calculated from all pixel temperatures within a single ROI and reflecting spatial temperature heterogeneity only; it does not represent repeated-measurement variability, uncertainty of the mean, camera precision, or technical repeatability. |ΔT| denotes the absolute difference between the mean temperatures of the right and left ROIs. At 68 h, only the remaining left skin island was assessed. Technical repeatability was not assessed.

Figure	Time [h]	Mean Temperature of the Left Skin Island ± Within-ROI SD [°C]	Mean Temperature of the Right Skin Island ± Within-ROI SD [°C]	|ΔT| [°C] (Right–Left)
Figure 4A,B	20	28.2 ± 0.6	28.8 ± 0.6	0.6
Figure 4C,D	28	31.5 ± 0.7	33.0 ± 0.5	1.5
Figure 4E,F	44	29.6 ± 0.7	30.0 ± 0.4	0.4
Figure 4G,H	49	32.7 ± 1.5	26.6 ± 1.8	6.1
Figure 4I	68	32.4 ± 0.9	--	--

**Table 4:** Serial Doppler ultrasonography findings and clinical assessment of the right and left skin islands during postoperative monitoring.

Time [h]	Clinical Assessment	Blind Doppler	Decision
20 h	Right and left skin islands were clinically viable, with no signs of skin-island compromise.	Audible signal present in both skin islands.	Observation; continuation of standard monitoring.
28 h	Right and left skin islands remained clinically viable. The right skin island showed a transiently higher surface temperature compared with the left skin island, without clinical signs of impaired viability.	Audible signal present in both skin islands.	Observation; continuation of standard monitoring.
44 h	Right and left skin islands remained clinically viable. The thermal difference between the skin islands decreased (ΔT 0.4 °C).	Audible signal present in both skin islands.	Observation; continuation of standard monitoring.
49 h	Left skin island remained clinically viable. Right skin island was pale, showed no bleeding after puncture, and did not demonstrate functional perforator perfusion.	No audible signal was obtained from the right perforator.	Reoperation and removal of the non-viable right skin island, based on clinical, Doppler and CTA findings.

## Data Availability

The data supporting the findings of this study are available from the corresponding author upon reasonable request, subject to ethical and privacy restrictions related to paediatric clinical data.

## Abbreviations

The following abbreviations are used in this manuscript:

AHI	Apnea–Hypopnea Index
ALT	Anterolateral thigh
CT	Computed tomography
CTA	Computed tomography angiography
DIRT	Dynamic infrared thermography
IACT	International Academy of Clinical Thermology
ICC	Intraclass correlation coefficient
ICG-FA	Indocyanine green fluorescence angiography
ICU	Intensive care unit
IRT	Infrared thermography
NETD	Noise-equivalent temperature difference
NIRS	Near-infrared spectroscopy
NS	Nager syndrome
PSG	Polysomnography
ROC	Receiver operating characteristic
ROI	Region of interest
SD	Standard deviation
TMJ	Temporomandibular joint
